# Prevalence of chronic kidney disease and its associated factors among diabetes mellitus patients in Dessie Referral Hospital, South Wollo, Ethiopia

**DOI:** 10.1038/s41598-024-59184-3

**Published:** 2024-04-22

**Authors:** Mohammed Adem, Wondyefraw Mekonen, Ahmed Ausman, Mohammed Ahmed, Ali Yimer

**Affiliations:** 1 Department of Biomedical Science, School of Medicine, College of Health Sciences, Woldia University, P.O. Box 400, Woldia, Ethiopia; 2 Department of Physiology, School of Medicine, Addis Ababa University, Addis Ababa, Ethiopia; 3 Department of Midwifery, College of Health Sciences, Samara University, Samara, Ethiopia; 4 Department of Public Health, College of Health Sciences, Woldia University, Woldia, Ethiopia

**Keywords:** Diseases, Medical research, Nephrology

## Abstract

Diabetes mellitus shares a large proportion of kidney failure. Despite many patients suffering from diabetes mellitus and its complications in Dessie City, no study was conducted in the study area that shows the prevalence and associated factors of chronic kidney disease among diabetes mellitus patients. Therefore, this study aims to assess the prevalence of chronic kidney disease and its associated factors among adult diabetes mellitus patients attending Dessie Referral Hospital, South Wollo, Northeast Ethiopia. An institutional-based cross-sectional study was conducted at Dessie Referral Hospital among 267 randomly selected adult diabetic patients. Data were collected using questionnaires administered by interviewers. The glomerular filtration rate was estimated from serum creatinine levels. Data were entered into Epi-data version 4.6 and analyzed using SPSS version 26 software. Multi-variable logistic regression was used to determine the strength of association for the associated factors of chronic kidney disease. Variables with a *p* value < 0.05 were used to ascertain statistically significant associations. A total of 267 diabetic patients participated in this study. About 104 (39%) of the respondents were female and from the total, 133 (48.1%) were hypertensive. The overall prevalence of chronic kidney disease in this study was 31.5% (95% CI 25.3–37.1%). Being older (*p*-value = 0.003) and having hypertension (*p*-value = 0.043) were significant factors for chronic kidney disease among diabetes mellitus patients. This study found a high prevalence (31.5%) of chronic kidney disease among diabetic patients. Older age, having hypertension, and elevated serum creatinine were statistically significant associated factors of chronic kidney disease among patients with diabetes mellitus. Thus, clinicians should be aware of the high prevalence of chronic kidney disease in Dessie City. Moreover, emphasis should be given for old age and hypertension as contributing factors to the high prevalence in diabetic patients.

## Introduction

Chronic kidney disease (CKD) is defined as structural or functional abnormalities of the kidney that persist for at least 3 months and is manifested by the presence of kidney damage (frequently detected as persistent albuminuria or proteinuria) or decreased estimated glomerular filtration rate (eGFR) which is defined as less than 60 ml/min per 1.73 m^2^^1^. The explanation of CKD is characterized by an extended asymptomatic period of nephropathy. Thus, early detection of renal impairments is vital to prevent or delay disease progression and reduce the risk of adverse outcomes^2^.

CKD has an impact on the kidney (kidney failure, progression of kidney disease, and acute kidney injury), as well as on other systems of the body (CVD, cognitive decline, anemia, micronutrient deficiency, and bone disorders^3,4^. Furthermore, CKD-affected people have significantly higher rates of morbidity, hospitalization, healthcare utilization, and premature death^5^. CKD is a worldwide public health problem affecting millions of people around the world^6^.

Diabetes Mellitus (DM), particularly type 2 diabetes, is the leading cause of CKD in both developed and developing countries^7,8^. Globally, more than 50% of type 2 DM patients developed CKD^9^. CKD is around 3–4 times more common in Africa than in the developed world^10^. In Ethiopia, like other developing countries, both the prevalence of DM and CKD are increasing rapidly^11,12^. For instance, the prevalence of DM in Dessie town (northeast Ethiopia) was 6.8%^13^. In the same way, the prevalence of CKD among DM patients is increasing, which ranges from 7.7 to 65.1%^11,14^. A systematic review of the literature conducted in 2020 shows that the prevalence of CKD among patients with DM in Ethiopia was found to be 35.52%^15^. A recent study conducted at Tibebe Ghion Specialized Hospital, Bahir Dar, shows that the prevalence of CKD was found to be 16.7% by using CKD EPI equations^10^. A study conducted in Northwest Ethiopia at the University of Gondar Hospital shows that the prevalence of CKD is 17.3% and 14.3% by the MDRD and CKD EPI equations, respectively^16^.

Another study conducted in 2016 at Dessie Referral Hospital (DRH) showed that the overall prevalence of CKD was 26.3%^17^. Though several studies have used the Cockcroft-Gault equation to assess the prevalence of CKD from eGFR in Ethiopia, our study has followed the recently applied CKD epidemiology equation (CKD-EPI 2009) to assess the prevalence of CKD among Diabetic patients.

In addition, many physicians in Ethiopia rely only on scr as a measure of renal function, and currently kidney failure and death due to CKD and its complications have become common^18^. Though many patients are suffering from DM (6.8%)^13^, there is information gap about the prevalence of CKD and its associated factors among patients with DM in adult population. Therefore, this study aimed to assess the prevalence of CKD using the CKD-EPI 2009 equation (https://www.mdcalc.com/calc/3939/ckd-epi-equations-glomerular-filtration-rate-gfr) and proteinuria (dipstick) for the early detection of CKD and its associated factors among DM patients attending Dessie Referral Hospital (DRH), South Wollo, Northeast Ethiopia.

## Methods

### Study design, period, and area

An institution-based cross-sectional was conducted from January 15, 2020, to February 5, 2020, at Dessie Referral Hospital, South Wollo, Northeast Ethiopia. Dessie town is located 401 km away from Addis Ababa, the capital city of Ethiopia.

### Study population

All adult DM patients attending at Dessie Referral Hospital were the source population and all adult DM patients (age ≥ 18 years) who were attending at the Hospital during the data collection period and who were willing to participate in the study were included as a study population.

### Exclusion criteria

Patients who were pregnant, known to have liver disease (cirrhosis), hospitalized, febrile, and had marked muscle wasting were excluded from the study.

### Sample size determination and sampling procedure

The sample size was calculated using the single population proportion formula with the following assumptions; 95% confidence level, 5% margin of error and the prevalence of CKD among patients with DM at Gondar Tertiary Hospital, Northwest, Ethiopia^19^, which was 21.8% and the final sample size became 267. Study subjects were selected using a systematic random sampling technique.

### Study variables

The outcome variable for this study was the prevalence of CKD (Yes or No) among patients with DM and the independent variables were sociodemographic factors (age, sex, marital status, educational level of respondents, income per month) and clinical and behavioral factors (hypertension, alcohol consumption, smoking, types of diabetes, duration of diabetes, systolic blood pressure, diastolic blood pressure, body mass index (BMI), fasting blood glucose, and blood urea level).

### Data collection tools and procedures

Two professional nurses using interviewer-administered questionnaires, anthropometric, and biochemical (laboratory) measurements collected data. The investigators played a supervisory role. The questionnaire included sociodemographic characteristics, such as age, sex, educational level, and income per month. It also contains clinical and behavioral factors for CKD in patients with DM such as hypertension, alcohol consumption, cigarette smoking, Types of diabetes, and duration of diabetes^19,20^.

### Anthropometric measurement

Physical measurements such as weight, height, BMI, and blood pressure were taken using standardized methods and adjusted equipment. The weight was measured in kg with light clothing and no wearing of shoes. The height was measured in centimeter using a vertical scale without shoes and in an upright position then, BMI was calculated as weight divided by height squared (kg/m^2^) and values of BMI was classified as follows: BMI ≤ 18.5 kg/m^2^ underweight, BMI = 18.5–24.9 kg/m^2^ normal weight, BMI = 25–29.9 kg/m^2^ overweight and BMI ≥ 30 kg/m^2^ obese.

Blood pressure was measured according to WHO guidelines using an automated sphygmomanometer after participants rested for at least five minutes or 30 min for those taking hot drinks like coffee. The sphygmomanometer cuff was normally placed smoothly and snugly on the upper arm. The arm is flexed at the heart level, while the subject is seated with the arm supported. The cuff is inflated until the radial pulse disappears. As the valve is gradually opened, the pressure of the cuff (slowly) decreases. When the cuff pressure is equal to the arterial systolic pressure, blood begins to flow through the cuff; audible sounds are heard using a stethoscope. Thus, the first knocking sound (Korot koff) was the subject's systolic pressure, and when the knocking sound disappeared, that was the diastolic pressure (such as 120/80). Hypertension was defined as mean blood pressure ≥ 140/90 mmHg or medical history of hypertension.

### Biochemical/laboratory tests

After collecting about 5 ml of blood sample (at least after eight hours of fasting) by the laboratory technologist with the standard venipuncture technique; serum glucose, serum creatinine and blood urea levels were quantified from it. Serum creatinine (Scr) was estimated using the alkaline picrate (“Jaffe”) method using a commercially available kit.

Freshly voided urine was also collected with clean and dry containers and the dry-reagent test strip technique (dipstick) was used for qualitative and semi quantitative estimation of proteinuria. The presence of albuminuria (+ 1 or more) in urine was defined as proteinuria. eGFR was calculated from serum creatinine (Scr) using the formula (CKD-EPI 2009) and CKD was defined as eGFR < 60 ml/min/1.73 m^2^ and/or albuminuria.

### Data quality control

To ensure the quality of the data, prior to data collection, the principal investigator conducted data collection training. The data collection tool was prepared in English and then translated into Amharic. The questionnaire was pretested in 15 patients at Tikur Anbessa Specialized Hospital (TASH) before proceeding with the research participants to check the validity and appropriateness of the questions included there. To ensure the quality of laboratory analysis, the standard operating procedures (SOPs) of the DRH clinical chemistry laboratory were strictly followed. Laboratory test data collectors were professional laboratory technologists and nurses under the close supervision of the investigator. Thus, all tests were standardized and automated, and all variables were filled in the data extraction format daily.

### Data processing and analysis

After the data was collected, it was properly coded and entered in the Epi-data version 4.6 (EpiData Software—http://www.epidata.dk) and then exported to a statistical package for the social science (SPSS) version 26 software (Downloading IBM SPSS Statistics 26) for analysis. Bi-variate and multi-variable logistic regression analysis was used to determine the potential determinants of risk factors for CKD among DM patients and a *p*-value of < 0.05 with a 95% confidence level was used to declare a significant statistical association.

### Ethical considerations and consent for participants

Ethical approval for the research was obtained from Addis Ababa University, College of Health Sciences, and School of Graduate Studies, Department of Medical Physiology Research Ethics Review Committee. A written permission letter was obtained from hospital managers. Written informed consent was obtained from the participants and from their guardians for those who are illiterate. Confidentiality was maintained by omitting direct personal identifiers on the questionnaire, using code numbers, storing data locked with a password, and not misusing or wrongfully disclosing their information. Participants were also informed that participation was voluntary and that they could withdraw from participation in the study at any stage if they were not comfortable with the investigation. Written informed consent was obtained from the study participants prior to starting the study.

## Results

### Sociodemographic characteristics

A total of 267 DM patients (163 males and 104 females) participated in this study. Concerning age, 204 (76.4%) of the sample were found in the age group of less than 60 years. Regarding marriage, 233 (87.3%) of the samples were married and 71 (26.6%) did not attend formal education (Table 1).

**Table 1:** Sociodemographic characteristics of the study participants (n = 267) in DRH, South Wollo, Northeast Ethiopia, 2020.

Characteristics	Total N	(%)
Age (year)		
18–60	204	76.4
> 60	63	23.6
Sex		
Male	163	61
Female	104	39
Marital status		
Married	233	87.3
Single	18	6.7
Divorced	2	0.7
Windowed	14	5.2
Educational status		
Illiterate	71	26.6
Primary school	64	24.00
Secondary school	68	25.5
Collage/university	40	15.0
Post graduate	24	9.0
Religion		
Orthodox	101	37.8
Muslim	124	46.4
Protestant	36	13.5
Other	6	2.2
Residence		
Urban	187	70
Rural	80	30
Monthly income		
≤ 1000 ETB	50	18.7
1001–2000 ETB	80	30.0
2001**–**3000 ETB	63	23.6
3001–4000 ETB	50	18.7
≥ 4000 ETB	24	9.0
Occupation		
Merchant	74	27.7
Housewife	30	11.2
Government employed	42	15.7
Self-employed	43	16.1
Farmer	50	19.7
Other*	28	10.5

*ETB* Ethiopian Birr, *N* Number, * = student.

### Behavioral and clinical characteristics of the participants

The study shows that 92.1% and 94.4% of the study participants never smoked cigarettes and never drank alcohol, respectively (Table 2).

**Table 2:** Behavioral and clinical characteristics of the study participants (n = 267) in DRH, South Wollo, Northeast Ethiopia, 2020.

Characteristics	Total N	(%)
Smoking status		
Yes	21	7.9
No	246	92.1
Alcohol drinking status		
Yes	15	5.6
No	252	94.4
BMI (Kg/m^2^)		
Underweight (< 18.5)	4	1.5
Normal weight (18.5–24.9)	71	26.6
Overweight (25–29.9)	60	22.5
Obese (≥ 30)	132	49.4
SBP (mmHg)		
< 140	90	33.7
≥ 140	177	66.3
DBP (mmHg)		
< 90	186	69.7
≥ 90	81	30.3
HTN		
Yes	133	49.8
No	134	50.2
FBG		
< 150 mg/dl	103	38.6
≥ 150 mg/dl	164	61.4
Types of DM		
Type 1	20	7.5
Type 2	247	92.5
Duration of DM		
< 5 years	137	51.3
5–10 years	94	35.2
≥ 10 years	36	13.5

*HTN* Hypertension, *FBG* Fasting blood glucose, *BMI* Body mass index, *SBP* Systolic blood pressure, *DBP* Diastolic blood pressure, *BUN* Blood urea nitrogen, *SCr* Serum creatinine, *N* Number.

Regarding nutritional status, 71 (26.6%) of the study participants had a normal BMI. High SBP and normal DBP were reported among 177 (66.3%) and 186 (69.7%) of the participants, respectively. About 133(49.8%) of the study participants were hypertensive. Regarding the type of DM, the majority of study participants (92.5%) were type—2 DM patients (Table 2).

### Renal function tests of study participants

As renal function tests, ${s}_{cr},$ BUN, proteinuria (dipstick) and eGFR (using CKD-EPI) were performed for all study participants. The mean (± SD) of SCr was 1.1(± 0.23) mg/dl. Similarly, the mean (± SD) of BUN was 25.3(± 4.1) mg/dl. Regarding eGFR, the mean (± SD) was 80.23 (± 25) ml/min/1.73 m^2^ using the CKD-EPI equation. Among the patients diagnosed with CKD, 45 (16.8%) had proteinuria (Table 3).

**Table 3:** kidney function tests of study participants at DRH, Wollo, South Northeast Ethiopia, 2020.

Parameter	Mean ± SD
SCr(mg/dl), Mean ± SD	1.1 ± 0.23
BUN (mg/dl), Mean ± SD	25.3 ± 4.1
eGFR CKD-EPI (ml/min/1.73 m^2^), Mean ± SD	80.23 ± 25.44

Parameter	Total N (%)
SCr	
≤ 1.2 mg/dl	170 (63.67%)
> 1.2 mg/dl	97 (36.32%)
BUN	
≤ 20 mg/dl	36 (13.5%)
> 20 mg/dl	231 (86.5%)
Proteinuria	
Positive	45 (16.9%)
Negative	222 (83.1%)

*SCr* Serum creatinine, *eGFR CKD-EPI* Estimated glomerular filtration rate using Chronic Kidney Disease-Epidemiological Collaboration, *SD* Standard deviation, *N* Number.

### The prevalence of CKD

The estimated total prevalence of CKD was 31.5% (95% CI 25.3–37.1%). Of all CKD patients, 4 (4.8%) were stage 1 (eGFR of ≥ 90 ml/min/1.73 m^2^), 12 (14%) were stage 2 (eGFR of 60–89.9 ml/min/1.73 m^2^), 67 (80%) were stage 3 (eGFR of 30–59.9 ml/min/1.73 m^2^) and 1 (1.2%) were stage 4 (eGFR of 15–29.9 ml/min/1.73 m^2^). Among patients with type 2 DM, 95.6% of the participants had renal impairment and albuminuria (Table 4).

**Table 4:** Prevalence and stage of CKD*.*

Stage	Description	eGFR (ml/min/1.73 m^2^) (by CKD-EPI)	N (%)
1	Normal eGFR with albuminuria	≥ 90	4 (1.5%)
2	Slightly decreased eGFR with albuminuria	60–89.9	12 (4.5%)
3	Moderately decreased eGFR	30–59.9	67 (25.08%)
3A	Mildly to moderately decreased eGFR	45–59.9	65 (24.34%)
3B	Moderately to severely decreased eGFR	30–44.9	2 (0.74%)
4	Severely decreased eGFR	15–29.9	1 (0.37)
5	Kidney failure	< 15	0
	Total		84 (31.5%)

*eGFR* Estimated glomerular filtration rate, *N* Number, *%* Percent.

**Figure 1:**
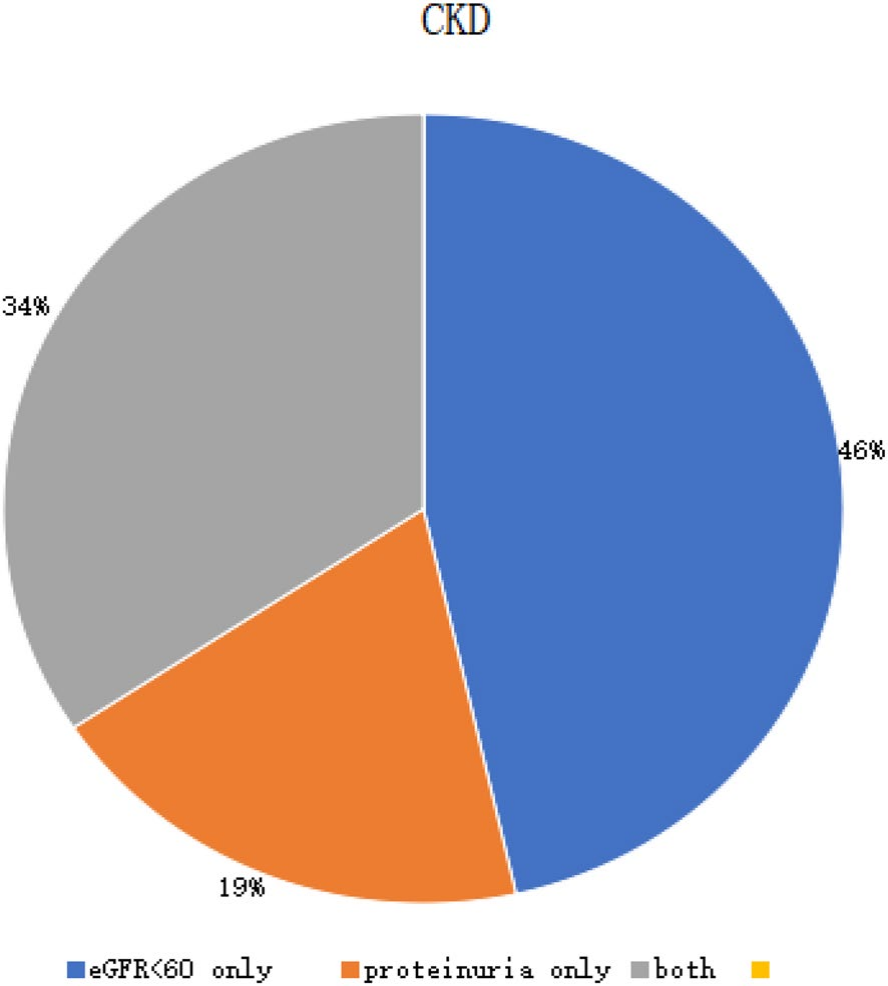
Pie chart showing the distribution of participants with CKD by renal impairment (eGFR < 60 ml/min/1.73 m^2^) and proteinuria.

Of these total CKD (84), 29 (34.22%) patients had both renal impairment (eGFR < 60 ml/min/1.73 m^2^) and proteinuria. 39 (46.42%) had only renal impairment (eGFR < 60 ml/min/1.73 m^2^) and 16 (19.04%) had only proteinuria (Fig. 1).

Of the total of study participants, 170 (63.67%) had normal S_cr_ (≤ 1.2 mg/dl). When these participants were assessed using the CKD-EPI formula, stage 3 CKD was found in 14 (8.23%) of these participants. (Table 5).

**Table 5:** Kidney function in DM patients with normal Scr assessed using CKD-EPI equations at DRH, South Wollo, Northeast Ethiopia,

GFR category (ml/min/1.73 m^2^)	Description	CKD-EPI N (%)
G1(≥ 90)	Normal or high GFR	86 (50.8%)
G2 (60–89)	Mildly↓GFR	70 (41.17%)
G3(45–59)	Mildly to moderately↓GFR	14 (8.23%)

*GFR* Glomerular filtration rate, *CKD-EPI* Chronic Kidney Disease-Epidemiological Collaboration, *N* Number.

### Risk factors for CKD

In the bivariate analysis, older age, obesity, elevated systolic blood pressure (SBP), elevated diastolic blood pressure (DBP), hypertension, longer duration of diabetes, elevated SCr, elevated BUN, and smoking habit were significantly associated with CKD (Table 6).

**Table 6:** Binary logistic regression analysis of patient characteristics associated with CKD at DRH, South Wollo, Northeast Ethiopia, 2020.

Variables	CKD by CKD-EPI		COR (95% CI)	*p*-value
	Yes	No		
	N (%)	N (%)		
Age (year)				
18–60	49 (18.4)	155 (58.1)	0.253 (0.140–0.457)	0.000*
> 60	35 (13.1)	28 (10.5)	1	
Sex				
Male	53 (19.9)	110 (41.2)	0.8881 (0.57–1.502)	0.642
Female	31 (11.6)	73 (27.3)	1	
Marital status				
Married	75 (28.1)	158 (59.2)	0.475 (0.161–1.402)	0.178*
Single	2 (0.7)	16 (6.0)	0.125 (0.21–0.7600)	0.024
Divorced	0 (0)	2 (0.7)	0.000 (0.0000)	0.999
Windowed	7 (2.6)	7 (0.6)	1	
Educational status				
Illiterate	25 (9.4)	46 (17.2)	0.906 (0.347–2.3641)	0.840
Primary school	18 (6.7)	46 (17.2)	0.652 (0.242–1.755)	0.397
Secondary school	49 (18.4)	19 (7.1)	0.646 (0.242–1.725)	0.383
Collage/university	27 (10.1)	13 (4.9)	0.802 (0.278–2.313)	0.684
Graduate	15(5.6)	9 (3.4)	1	
Residence				
Urban	60 (22.5)	127 (47.6)	1.102 (0.624–1.946)	0.737
Rural	24 (9.0)	56 (21.0)	1	
Monthly income				
≤ 1000 ETB	12 (4.5)	38 (14.2)	0.373 (0.133–1.048)	0.061*
1001–2000 ETB	17 (6.4)	63 (23.6)	0.819 (0.121–0.837)	0.020
2001**–**3000 ETB	27 (10.1)	36 (13.5)	0.886 (0.344–2.281)	0.803
3001–4000 ETB	17 (6.4)	33 (12.4)	0.609 (0.225–1.644)	0.328
≥ 4000 ETB	11 (4.1)	13 (4.9)	1	
Smoking status				
Yes	10 (3.7)	11 (4.1)	2.11 (0.860–5.190)	0.102*
No	74 (27.7)	172 (64.4)	1	
Alcohol drinking status				
Yes	7 (2.6)	8 (3.0)	1.989 (0.696–5.678)	0.199*
No	77 (28.8)	175 (65.5)	1	
BMI (Kg/m^2^)				
Underweight (< 18.5)	2 (0.7)	2 (0.7)	1.538 (0.210–11.264)	0.671
Normal weight (18.5–24.9)	14(5.2)	57 (21.3)	0.378 (0.91–0.747)	0.005*
Overweight (25–29.9)	16(6.0)	44 (16.5)	0.559 (0.286–1.094)	0.089
Obese (≥ 30)	52 (19.5)	80 (30)	1	
SBP (mmHg)				
< 140	16 (6.0)	74 (27.7)	0.347 (0.187–0.644)	0.001*
≥ 140	68 (25.0)	109 (40.8)	1	
DBP (mmHg)				
< 90	45 (16.9)	141 (52.8)	0.344 (0.198–0.596)	0.000*
≥ 90	39 (14.6)	42 (15.7)	1	
HTN				
Yes	54 (20.2)	79 (29.61)	2.370 (1.390–4.040)	0.002*
No	30 (11.2)	104 (39.0)	1	
FBG				
< 150 mg/dl	33(12.4)	70 (26.2)	1.042 (0.615–1.774)	0.872
≥ 150 mg/dl	51(19.1)	113 (42.3)	1	
Types of DM				
Type 1	5 (1.9)	15 (5.6)	0.709 (0.249–2019)	0.519
Type 2	79 (29.6)	168 (62.9)	1	
Duration of DM				
< 5 years	29 (10.9)	108 (40.4)	0.300 (0.139–0.650)	0.002*
5–10 years	38 (14.2)	56 (21.0)	0.483 (0.758–0.3500	0.483
≥ 10 years	17 (6.4)	19 (7.10)	1	

ETB: Ethiopian Birr; N: Number, *: *p*-value < 0.25.

After incorporating all the variables of significance (*p*-value < 0.25) variables in the bivariate logistic regression analysis, multivariable logistic regression analysis was performed to identify the risk factors independently associated with CKD. The multivariable logistic regression analysis showed that only older age, HTN, and elevated SCr were independently associated with CKD (Table 7).

**Table 7:** Multi-variable Binary Logistic Regression Analysis of Patient Characteristics associated with CKD among DM patients at DRH, Wollo, South Northeast Ethiopia ,2020.

Variables	CKD by CKD-EPI		AOR (95% CI)	*p*.-value
	Yes	No		
	N (%)	N (%)		
Age (year)				
≤ 60	49 (18.4)	155 (58.1)	0.262 (0.108–0.637)	0.003*
> 60	35 (13.1)	28 (10.5)		
Marital status				
Married	75 (28.1)	158 (59.2)	2.371(0.548–10.262	0.675
Single	2 (0.7)	16 (6.0)	0.773 (0.076–7.819)	0.827
Divorced	0 (0)	2 (0.7)	0.000	0.868
Windowed	7 (2.6)	7 (0.6)		
Monthly income				
≤ 1000 ETB	12 (4.5)	38 (14.2)	0.764 (0.217–2.6890	0.675
1001–2000 ETB	17 (6.4)	63 (23.6)	0.493 (0.147–1.6530	0.252
2001**–**3000 ETB	27 (10.1)	36 (13.5)	0.908 (0.259–2.8450	0.868
3001–4000 ETB	17 (6.4)	33 (12.4)	1.284 (0.382–4.318)	0.686
≥ 4000 ETB	11 (4.1)	13 (4.9)	1	
Smoking status				
Yes	10 (3.7)	11(4.1)	1.298 (0.403–4.190)	0.661
No	74 (27.7)	172 (64.4)	1	
Alcohol drinking status				
Yes	7 (2.6)	8 (3.0)	0.526 (0.119–2.330)	0.397
No	77 (28.8)	175 (65.5)	1	
BMI (Kg/m^2^)				
Underweight (< 18.5)	2 (0.7)	2 (0.7)	11.714 (0.712–192.767)	0.085
Normal weight (18.5–24.9)	14 (5.2)	57 (21.3)	0.669 (0.287–1.557)	0.351
Overweight (25–29.9)	16(6.0)	44 (16.5)	1.049 (0.465–2.365)	0.908
Obese (≥ 30)	52 (19.5)	80 (30)	1	
SBP (mmHg)				
< 140	16 (6.0)	74 (27.7)	0.674 (0.316–1.485)	0.306
≥ 140	68 (25.0)	109 (40.8)	1	
DBP (mmHg)				
< 90	45 (16.9)	141 (52.8)	0.535 (0.64–1.084)	0.083
≥ 90	39 (14.6)	42 (15.7)	1	
HTN				
Yes	54 (20.2)	79 (29.61)	2.279 (1.025–5.067)	0.043*
No	30 (11.2)	104 (39.0)	1	
Duration of DM				
< 5 years	29 (10.9)	108 (40.4)	0519 (0.199–1.352)	0.179
5–10 years	38 (14.2)	56 (21.0)	0.944 (0.357–2.494	0.908
≥ 10 years	17 (6.4)	19 (7.10	1	

ETB: Ethiopian Birr; N: Number, *: *p*-value < 0.05.

## Discussion

This study has assessed the prevalence and risk factors of CKD among diabetic adults at Dessie Referral Hospital using an estimated glomerular filtration rate (eGFR) and proteinuria (dipstick). The estimated total prevalence of CKD was 31.5% (95% CI 25.3–37.1%).

A similar study from the UK presented a similar CKD prevalence (31.5%)^21^. The finding of our study is also relatively similar to those observed in China (29.6%)^22^ and Spain 27.9%)^23^.

Our prevalence estimate of CKD was much lower than the study done in Asella that showed the overall prevalence of CKD was 86.3% by C–G equation ^14^. Possible reasons for this difference might be the variation in the sensitivity of the estimators used and the difference in the study design. The study^14^ used the C–G equation for the estimation of GFR, which underestimates the GFR, thereby increasing the prevalence of CKD. Different results showed that the prevalence of CKD and its staging can vary even within analogous populations depending on the estimators used^24^. The CKD-EPI equation has been advised to obtain a more accurate GFR in numerous African people^25^.

On the contrary, the estimated prevalence of CKD in our study was higher than that of studies conducted in Gondar (25.3%)^19^ and Butajira (23.8%)^26^. The possible reasons for this variation are differences in the mix of cases (in terms of age), sample size, and creatinine assays. In our sample, there are more aged participants compared to the previous two studies. Furthermore, the latter study (Butajira) used only eGFR to estimate the prevalence of CKD, which possibly reduced the prevalence of CKD.

However, the estimated prevalence of CKD in this study appeared low compared to most other studies conducted outside the country, which revealed a prevalence of CKD based on eGFR < 60 and ACR in the range of 34–86^27–32^. A cross-sectional study conducted in the USA also showed that the prevalence of CKD in patients with diabetes was found to be 39.7%^33^.

The lower prevalence in this study may be attributed in part to the fact that we did not use the ACR or microalbuminuria test. Furthermore, ethnic differences, sample size variation, and differences in sensitivity of the estimators used and lifestyle can contribute to the observed difference.

The prevalence of CKD in this study was approximately 3 times higher than that reported from Botswana (8.4%)^34^. This might be because they used only eGFR to estimate the prevalence of CKD, but in this study, we used both eGFR and proteinuria.

A similar institutional-based study conducted in 2016 at Dessie Referral Hospital (the same sitting as this one) showed the overall prevalence of CKD as 26.3%^17^. In this previous study, only 13% of the participants had CKD defined by eGFR < 60 ml/min/1.73 m^2^ using the MDRD equation, and the remaining (13.3%) had CKD defined by albuminuria using the urine dipstick test. The prevalence of CKD in this report using the MDRD equation is lower than the finding in our study, which is done by the CKD-EPI equation (26.3% vs. 31.5%). This could be due to differences in the case mix (in terms of age and being hypertensive) and the difference in the equation (formula) that we used to determine the eGFR. There are more hypertensive participants in our study than in the previous study, which is 133(49.8%). vs 99 (30.7%). In the same way, the numbers of aged participants in this study are more than in the previous study (23% vs. 14.9%).

Regarding risk factors, this study found a significant association between older age and CKD defined by CKD-EPI (*p* = 0.003) equation. This result is consistent with the findings from other studies^19,26,35,36^. In elderly patients, glomerular filtration may decrease as part of the body’s aging process^37^. This is because as age increases, there is a progressive loss of nephrons and decreased renal blood flow^38^. It means that as age increases, the GFR will decrease, causing a higher number of people with CKD in the older age groups. Thus, CKD screening in the elderly age group is an important strategy to improve outcomes.

This study also reveals that hypertension was independently associated with CKD (*p* = 0.043) defined by the CKD-EPI equation. This is consistent with other related studies^14,39,40^. Systemic hypertension is transmitted by intra glomerular capillary pressure leading to glomerulusclerosis and loss of kidney function^41^. Therefore, blood pressure control is a key component in preventing and slowing the progression of CKD.

Our study indicates that several participants, 14 (8.23%) of participants whose scr were in the normal range had mild to moderate RI (stage 3 CKD) indicating that the lower prevalence of impaired renal function observed through serum creatinine. This supports the literature findings that state serum creatinine alone is not a sensitive method to assess renal function in asymptomatic CKD patients. Thus, as stressed by current guidelines, it is recommended not to use (SCr) alone to assess kidney function, because it fails to identify many patients whose kidney function is reduced while they remain within the normal range^18,42^.

### Limitations of the study

The results of this study need to be interpreted with some limitations to put the findings into perspective. Albumin creatinine ratio is a better indicator in the detection of CKD, but, for feasibility reasons, we used semi quantitative methods (dipstick) to assess proteinuria. Only single serum creatinine and urine protein measurements were performed (instead of at least two serum levels performed three months apart), and therefore we could not distinguish those patients with temporary disorders in renal function disorders from those with true CKD. Furthermore, the influence of other medications and diets was also not considered during this study.

## Conclusion

The study revealed that the prevalence of CKD is high in the study area. Older age, the presence of HTN, and elevated serum creatinine were associated factors of the development of CKD in patients with DM. Therefore, clinicians in outpatient diabetes clinics should be aware of the high prevalence of CKD, and elderly people and patients with HTN should receive special attention, as they are more at risk of CKD. The renal insufficiency corresponding to participants in stage 3 CKD was also higher despite having normal scr levels, indicating that current screening methods (assessment of scr alone or urine dipstick for albuminuria) are decreasing the prevalence of CKD in DRH. Therefore, regular evaluation of renal function by eGFR and albuminuria (instead of scr alone) will help to recognize CKD at an early stage and to take an appropriate intervention.

## Data Availability

The results of this study were analyzed using the primary data collected. All relevant data are included in this research.

## Abbreviations

AOR: Adjusted odd ratio
BMI: Body mass index
CI: Confidence interval
CKD: Chronic kidney disease
DM: Diabetes mellitus
GFR: Glomerular filtration rate
eGFR: Estimated glomerular filtration rate
HTN: Hypertension
SPSS: Statistical package for social sciences
WHO: World Health Organization
TASH: Tikur Anbessa Specialized Hospital
SOPs: Standard operating procedures
MDRD: Modification of diet in renal disease
CKD-EPI: Collaboration of chronic kidney disease epidemiology collaboration
ACR: Albumin–creatinine ratio
